# GlycoMME, a Markov modeling platform for studying N-glycosylation biosynthesis from glycomics data

**DOI:** 10.1016/j.xpro.2023.102244

**Published:** 2023-04-21

**Authors:** Chenguang Liang, Austin W.T. Chiang, Nathan E. Lewis

**Affiliations:** 1 Department of Pediatrics, University of California, San Diego, La Jolla, San Diego, CA 92130, USA; 2 Department of Bioengineering, University of California, San Diego, La Jolla, San Diego, CA 92130, USA

**Keywords:** Bioinformatics, Biotechnology and bioengineering, Systems biology

## Abstract

Variations in N-glycosylation, which is crucial to glycoprotein functions, impact many diseases and the safety and efficacy of biotherapeutic drugs. Here, we present a protocol for using GlycoMME (Glycosylation Markov Model Evaluator) to study N-glycosylation biosynthesis from glycomics data. We describe steps for annotating glycomics data and quantifying perturbations to N-glycan biosynthesis with interpretable models. We then detail procedures to predict the impact of mutations in disease or potential glycoengineering strategies in drug development.

For complete details on the use and execution of this protocol, please refer to Liang et al. (2020).^1^

## Highlights

• GlycoMME enables easy visualization of *N*-linked glycosylation from glycomics data
• GlycoMME constructs models of glycan biosynthesis based solely on glycomics data
• GlycoMME provides biological insights on N-glycosylation with interpretable parameters
• GlycoMME can facilitate glycobiology studies by predicting GT-knockout glycoprofiles

## Abstract (editor highlights)

Publisher’s note: Undertaking any experimental protocol requires adherence to local institutional guidelines for laboratory safety and ethics.

## Graphical abstract

**Figure:**
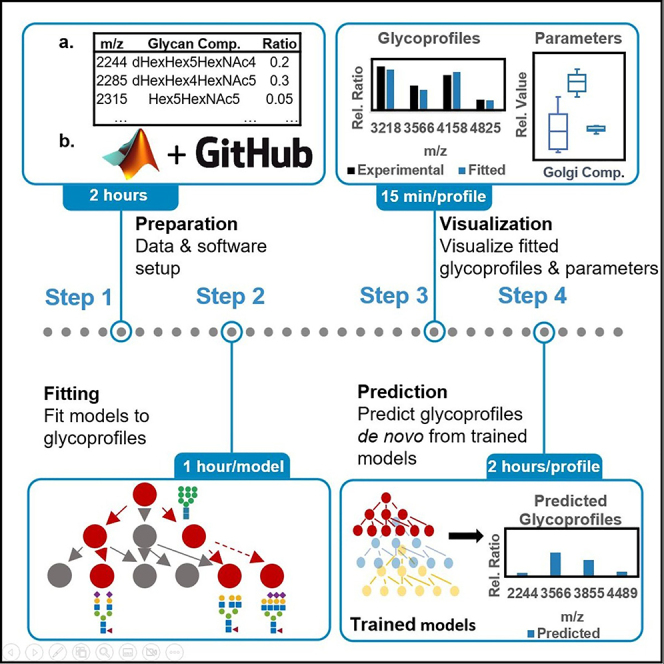

## Before you begin

### Overview

**Timing: variable (<45 min with preprocessed data; <5 min if MATLAB and relevant toolboxes have been pre-installed). Additionally, undetermined amounts of time will be needed for data curation depending on the total numbers of glycoforms or signals in users’ glycomic datasets, the curators’ familiarity with LinearCode, and any additional steps to normalize and organize glycomics data into the required matrix form as shown in the *Data.xlsx* file.**

Glycans coat most cells and decorate most proteins a cell uses to interact with its environment.^2^^,^^3^^,^^4^ Importantly, these glycans frequently modulate protein-protein interactions and participate in self-non-self recognition.^5^^,^^6^ Small changes in glycan structure can result in major impacts on protein function and organismal phenotypes.^5^^,^^7^ Thus, there is particular interest in understanding the genetic basis of any changes in glycosylation. Furthermore, the modulation of glycosylation on therapeutic proteins can increase potency and safety, and biosimilar development requires the achievement of glycans that are equivalent to the innovator drug.^8^^,^^9^^,^^10^^,^^11^ However, glycans are complex and their control is challenging since they are synthesized in complex pathways. Thus, there is a need to develop computational models of glycosylation to enable the use of systems glycobiology to control glycan structures.^12^

Many types of models have been constructed for glycosylation.^13^^,^^14^^,^^15^^,^^16^^,^^17^^,^^18^^,^^19^ While many require the enumeration of large numbers of kinetic parameters, which are difficult to obtain, more recently a different approach has been taken wherein glycan biosynthesis is modeled as a Markov process,^1^^,^^16^^,^^20^ wherein only a glycoprofile is needed for a sample of interest to parameterize a detailed and comprehensive model of glycosylation. Such models show great potential in being used to guide the glycoengineering of cells to obtain desired glycoprofiles on therapeutic proteins and biosimilars.^16^ They could further provide insights into the genetic basis of changes in glycosylation seen in diverse diseases. Here we present a protocol describing GlycoMME, a modeling toolbox that makes use of the Markov modeling principles.

This section includes minimal hardware requirements, installation process, and the files required for the pipeline.

### Download GlycoMME toolkit

**Figure 1:**
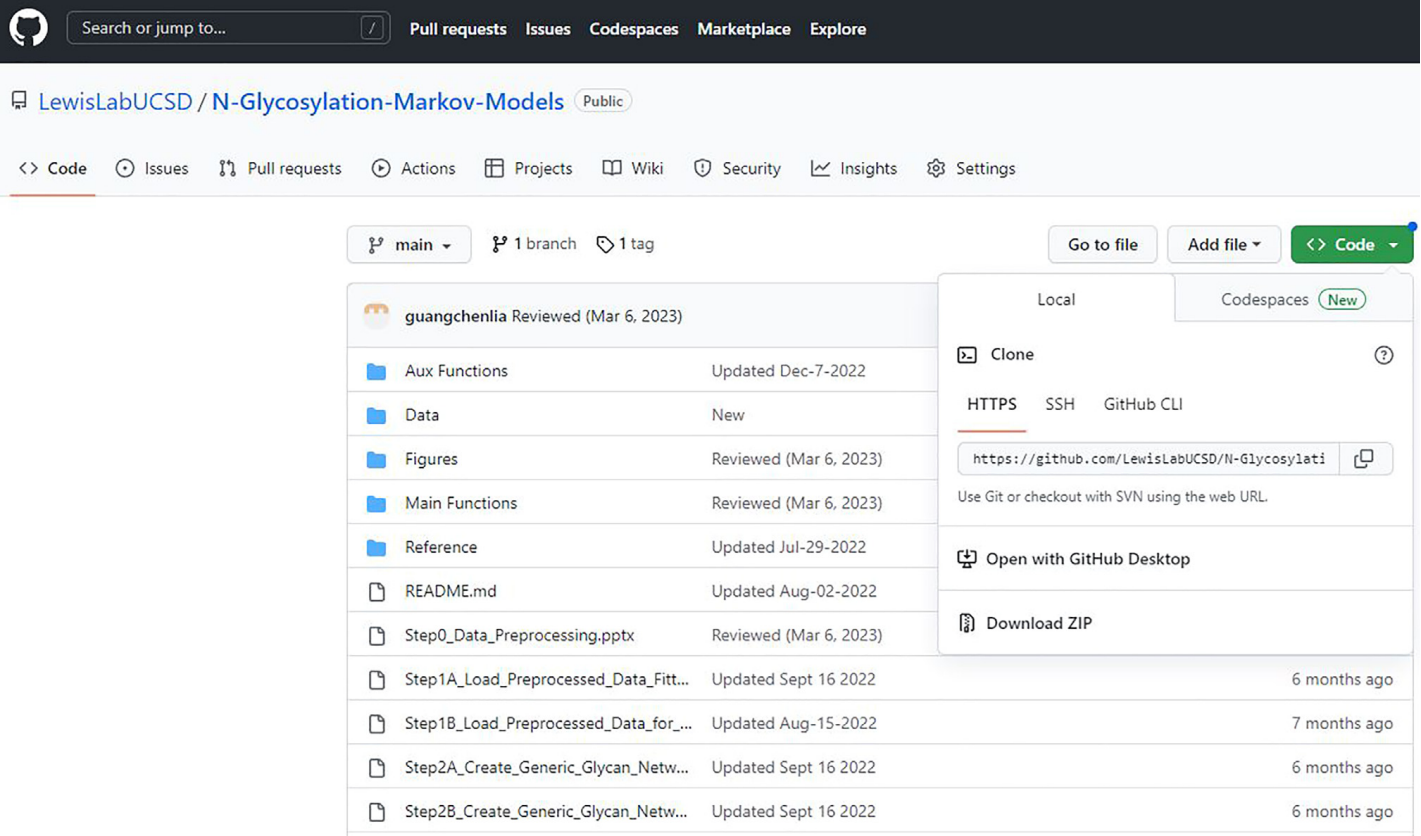
Screenshot of the GitHub page from which the toolkit can be downloaded
2. Unzip everything into the same master folder (default name as *N-Glycosylation-Markov-Models-main*).

***Note:*** In the master folder, everything in the *Data* folder (including those in the *OptimizationResults* subfolder), except for *Data.xlsx* and *Data_user.xlsx*, should be cleared or stored in another folder if users intend to use their own datasets.

1. Download GlycoMME toolkit from https://github.com/LewisLabUCSD/N-Glycosylation-Markov-Models (Figure 1). Click the green “Code” button at the upper right corner and download the toolkit and the example dataset as a zip file by clicking “Download Zip”.

### Initiate working environment in MATLAB

Users must obtain and install MATLAB beforehand. Once installed, choose the master folder as the “Current Folder” if the master folder is not the “Current Folder”:

3. Click and open MATLAB.
4. In MATLAB, click the icon in the red square (Figure 2A).

**Figure 2:**
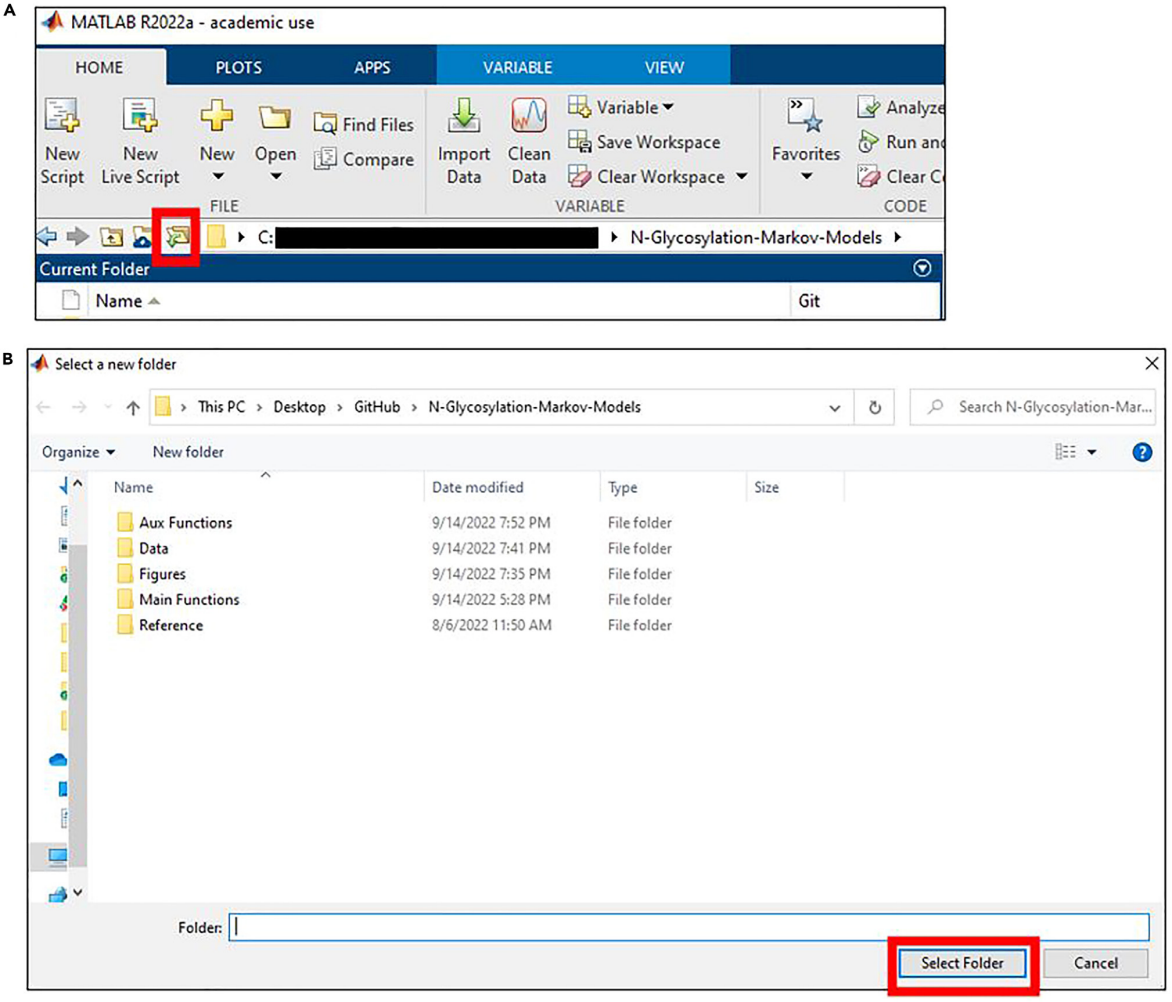
Set up the working environment in MATLAB (A) Click the icon in the red square in MATLAB. (B) Navigate to the master folder and click “Select Folder”.
5. Navigate to the master folder (default name:*N-Glycosylation-Markov-Models-main*) and click Select Folder (Figure 2B).

### Data collection

***Note:*** Glycomics data can be collected in different forms and some third-party glycomics data may have incomplete information regarding the glycoforms. For ease of manipulation, glycomics data should be organized in the provided excel sheet *Data_user.xlsx* (listed in key resources table). An example properly formatted dataset (*Data.xlsx*) is provided in the *Data* folder for reference.

6. Raw glycomic data need to be pre-processed before running the pipeline. Depending on the data types, two scenarios are possible:
  a. Glycomics data with known glycan composition (monosaccharides), with or without glycan structure annotations
    i. Open *Data_user.xlsx* in the *Data* folder and select the sheet “MS Raw”.
    ii. Enter in the first column all the m/z values at which glycans are detected. Each m/z value should be unique.
    iii. Enter in the second column all the glycan compositions corresponding to the m/z values in the previous step.
      ***Note:*** refer to *Step0_Data_Preprocessing.pptx*, slide 1, for the format of the strings used to represent the glycan compositions.
    iv. Starting from the third column, enter the relative MS signals (“areas under the curve”) corresponding to the m/z values.
      ***Note:*** Each column represents the glycomic profile of one sample and the top cell of each column should be the sample name. The sample names should only contain alphanumeric characters and forward slashes. For glycosyltransferase knockout predictions, the sample name must be “WT” for the base or wildtype glycomic profile, and symbols of knocked-out glycosyltransferases separated by forward slashes. If the same glycan composition corresponds to multiple m/z values (artifacts of different fragmentation patterns or ionization), the signals should be combined, and the smallest m/z value should be used.
    v. If glycan structure annotations are available, select the sheet “Annotation”
    vi. In the second column, enter all the glycan annotations as strings of Linear Code. Each glycan annotation should be unique. However, glycan annotations of different glycoforms at the same m/z value are allowed.
      ***Note:*** Linear Code^21^ is the format utilized by the pipeline to represent glycan structure annotations. For users unfamiliar with linear codes, we included a reference list of linear codes for common *N-*glycans and their corresponding graphical representations. The lists can be found in *Step0_Data_Preprocessing.xlsx*, slide 2–4 in the master folder.
    vii. Enter in the first column the m/z values corresponding to the annotations.
    viii. For each element of a column (excluding the sample name), enter 1 if the specific annotation (row) is present in the glycomic profile of the specific sample (column), and enter 0 otherwise. The sample names should be identical to those in sheet “MS Raw”.
      ***Note:*** Starting from the third column, each column represents a sample and the top cell of each column is a sample name.
  b. Known glycan structure annotations but without glycan compositions
    i. Open *Data_user.xlsx* in the *Data* folder and select the sheet “MS Raw”.
    ii. Similar to step 6a, vii, enter in the second column all the glycan annotations as strings of Linear Code. Each glycan annotation should be unique.
    iii. For each element of a column (excluding the sample name), enter the relative abundance for each specific glycan structure annotation (row) in each glycomic profile of a sample (column). Otherwise enter 0.
      ***Note:*** Starting from the third column, each column represents a sample and the top cell of each column is a sample name. The sample names should only contain alphanumeric characters and forward slashes. For glycosyltransferase knockout predictions, the sample name must be “WT” for the base or wildtype glycomic profile, and symbols of knocked-out glycosyltransferases separated by forward slashes.
    iv. Open and run the script *Sup1_Get_compositions_from_linearcodes.m* in the master folder to automatically populate the datasheet *Data_user.xlsx.*
      Once completing data pre-processing, users may compare the format of *Data_user.xlsx* with that of *Data.xlsx* as a quality check.

## Key resources table

**Table:** 

REAGENT or RESOURCE	SOURCE	IDENTIFIER
**Deposited data**		

Data.xlsx	(Yang et al.^22^)	https://github.com/LewisLabUCSD/N-Glycosylation-Markov-Models/blob/main/Data/Data.xlsx
DrugXData.xlsx	(Liang et al.^1^)	https://github.com/LewisLabUCSD/N-Glycosylation-Markov-Models/blob/main/Data/DrugXData.xlsx
Data_user.xlsx	This article	https://github.com/LewisLabUCSD/N-Glycosylation-Markov-Models/blob/main/Data/Data_user.xlsx
GlycoMME Toolkit master folder	This article	https://github.com/LewisLabUCSD/N-Glycosylation-Markov-Models

**Software and algorithms**		

MATLAB v.2020b	MathWorks	https://www.mathworks.com/products/matlab.html
Operators and Elementary Operations	MathWorks	Accompanied with MATLAB v.2020b
Global Optimization	MathWorks	Accompanied with MATLAB v.2020b
Parallel Computing	MathWorks	Accompanied with MATLAB v.2020b
Statistics and Machine Learning	MathWorks MathWorks	Accompanied with MATLAB v.2020b
Econometrics	MathWorks	Accompanied with MATLAB v.2020b
Curve Fitting	MathWorks	Accompanied with MATLAB v.2020b
System Identification	MathWorks	Accompanied with MATLAB v.2020b
Graph and Network Algorithms	MathWorks	Accompanied with MATLAB v.2020b

**Other**		

AMD Ryzen 7, 16 GB x2	AMD	https://www.amd.com/en/processors/ryzen-processors-laptop?utm_source=bing&utm_medium=cpc&utm_campaign=US%7CCONS%7CBP%7CRyzen&utm_term=Ryzen&utm_content=Ryzen_Phrase

## Step-by-step method details

Here, the described step-by-step methods are for two purposes as demonstrated in Liang et al.^1^ First, a generic Markov model is fitted to the glycomic profiles (glycoprofiles) of a glycoprotein drug produced by different glycoengineered cell lines. By visualizing the biosynthetic models of *N*-glycosylation, users gain insights into the glycoengineering impact on theoretical glycosylation reactions. Second, one may use the fitted models of single-glycosyltransferase-knockout (single-GT-KO) cell lines to predict the theoretical glycoprofiles of a glycoprotein produced by the cell lines with combinatorial GT knockouts. The glycoprotein whose glycoprofiles are predicted can be a different protein from the glycoprotein whose single-GT-KO glycoprofiles are used for fitting. As examples, we generated sample-specific models for 7 glycoprofiles of glycoengineered erythropoietin (EPO) produced by different single-GT-KO CHO-K1 or wildtype cell lines.^22^ We then use a few fitted single-GT-KO EPO models to predict 2 glycoprofiles of EPO produced by the cell lines with combinatorial glycosyltransferase (GT) knockouts. Lastly, we use a few fitted single-GT-KO EPO models to predict 2 glycoprofiles of Enbrel produced by similar CHO cell lines with the knockout impact learned from the EPO models.

### Visualize and preprocess dataset

**Timing: 5 min**

This step prepares the data in the *Data_user.xlsx* for usage by MATLAB and visualizes the user-supplied experimental glycoprofiles (glycomic data) and glycan structure annotations (if available). This step can also be used to check the quality of entered data.

1. After initiating the MATLAB working environment, open the MATLAB script *Step1A_Load_Preprocessed_Data_Fitting.m.*
2. In the script editor, specify the name of the dataset to be used at line 5.

***Note:*** “Data.xlsx” is the name for the demonstration dataset and “Data_user.xlsx” is the name for the user-supplied dataset.

**Figure 3:**
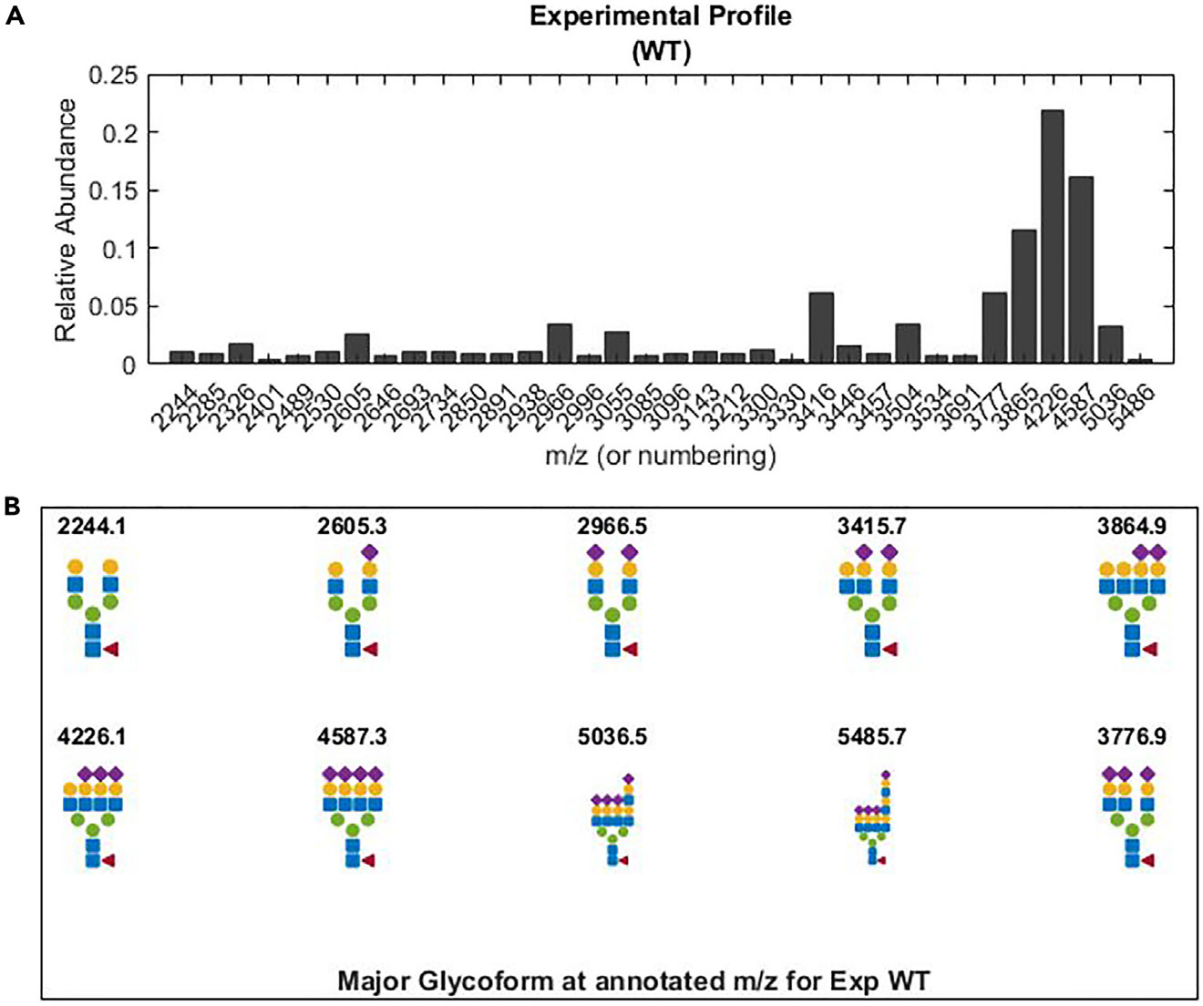
Visualization of the experimental glycoprofile of EPO produced by the wildtype CHO-K1 (A and B) (A) relative glycomic profile (B). glycan structures for each annotated m/z values, if provided. (B) will not be generated if no annotation is provided.

***Note:*** for advanced users, rendering selected glycan annotations only can be achieved by modifying the inputs of function *visualizeExpData* according to the comments in the script.

> DatafileName = 'Data.xlsx';

3. Run the entire script. A figure of the relative glycomic signals and a figure of glycan annotations will be generated for each sample, such as shown in Figure 3.

4. The pre-processed data are stored in the structure variable *DataSet* and as MATLAB-readable file *Data.mat* in the *Data* folder.

***Note:*** for advanced users, information regarding the created variables for pre-processed data can be found as comments in the script.

### Construct a generic *N*-glycosylation Markov model

**Timing: 10 min**

This step will generate a generic *N*-glycosylation Markov model, which will be later used to generate sample-specific models by fitting to the experimental glycoprofiles.

5. Open the MATLAB script *Step2A_Create_Generic_Glycan_Network_Fitting.m.*
6. In the script editor, specify the name of the dataset to be used in line 4.

> load Data.mat

***Note:*** “Data.xlsx” is the name for the demonstration dataset and “Data_user.xlsx” is the name for the user-supplied dataset.

7. Run the entire script.

***Note:*** for advanced users, the scope of the reactions considered can be achieved by modifying the variable *RxnSel* and the inputs of function *CreateGlycanRxnList* according to the comments in the script. For practicality, the complexity level of the network should not exceed 23. Complexity level is defined as the maximal number of times a core Man9 glycan is modified by glycosidases and/or glycosyltransferases in a stepwise fashion.

8. The generic model and fitting parameters are stored in the structure variable *GenericNetwork* and as MATLAB-readable file *GenericNetwork.mat* in the *Data* folder.

### Generate sample-specific *N*-glycosylation Markov models by fitting to experimental glycoprofiles

**Timing: 40 h (1 h/fitted model × 15 fitted models per sample (or 30 fitted models for the WT sample) × 7 samples / 3 MATLAB sessions)**

This step generates sample-specific *N*-glycosylation Markov models by fitting the generic model with their corresponding experimental glycoprofiles. This is a time-intensive step, and multiple MATLAB sessions can run in parallel to expedite the process.

9. Open the MATLAB script by clicking on *Step3A_Fit_Markov_Models_with_WT_Glycoprofile.m* in the “Current Folder” panel in MATLAB.
10. In the script editor, specify the name of the dataset to be used in line 4.

> load Data.mat

***Note:*** “Data.xlsx” is the name for the demonstration dataset and “Data_user.xlsx” is the name for the user-supplied dataset.

11. Specify the name(s) of the samples (glycoprofiles) to be fitted in line 13. For example

> ProfSel =

{'WT','B4galt1','St3gal3','St3gal4','St3gal6','B3gnt2','Mgat2'};

***Note:*** The name(s) provided here should be the same as the names appearing in the variable *DataSet.ProfNames*. To fit all the profiles in the dataset, users can instead specify the variable *ProfSel* as:

> ProfSel = DataSet.ProfNames;

12. Specify the number of models to be fitted for the wildtype sample (base glycoprofile) in Line 25 and for each of the other samples in Line 23. For Example:

> num = 5; % Number of models fitted for each profile

> if strcmp(ProfSel{a},'WT')

 num = 10; % Number of models fitted for the WT profile

> end

***Note:*** this is the number of models fitted for each profile per MATLAB session. In this case, 3 MATLAB sessions were run in parallel, with a total of 5 × 3 = 15 models fitted for each single-GT-KO profile and 10 × 3 = 30 models fitted for the wildtype profile.

13. Specify any arbitrary number for *IDNum* in Line 97. For example:

> IDNum = 1;

> savedFileName =

 ['OptimizationResults_',num2str(IDNum),'.mat'];

***Note:*** The fitted model parameters are stored in the structure variable *OptimizationResults* and as MATLAB-readable file *OptimizationResults_#.mat* in the *Data/OptimizationResults* folder. Here, number 1 is arbitrarily chosen as the *IDNum* and *OptimizationResults_1.mat* will be the file name.

14. Run the script.

***Note:*** After step 14, users may decide whether to run multiple MATLAB sessions in parallel to reduce the time needed to obtain sufficient models for each sample. The memory-intensive optimization process will need approximately 8 GB RAM per MATLAB session. The number of fitted models per hour will be approximately equal to the number of MATLAB sessions. 3 MATLAB sessions were run in parallel for this demonstration. The amount of RAM (% usage) used per MATLAB session can be obtained from the operating system’s task manager while running one session when using the parallel pool in MATLAB. To ensure fitting quality, MATLAB sessions should not collectively utilize >95% RAM.

***Optional:*** To run a new MATLAB session in parallel, open a new MATLAB session and repeat steps 9–14. It is essential to specify a unique *IDNum* number at step 13 for each MATLAB session so that the saved files *OptimizationResults_#.mat* from the different sessions do not overwrite each other.

**Figure 4:**
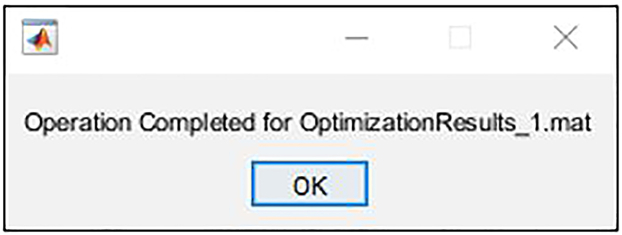
A message box will appear when fitting is completed in a MATLAB session

15. A message box (Figure 4) will show up when the fitting of all the glycoprofiles in a MATLAB session is completed. If multiple MATLAB sessions are opened, close all but one MATLAB session when all runs are completed.

### Visualize fitted glycoprofiles from sample-specific models

**Timing: 2 h (approx. 15 min per sample)**

This step generates figures showing the quality, parameter values (transition probabilities), and other characteristics of the fitted models and glycoprofiles, in comparison to the experimental profile. This step also stores computed features of the fitted models, which are used for prediction purposes in later steps.

16. Click on *Step4A_Visualize_Fitted_Glycoprofiles.m* in the “Current Folder” panel in MATLAB.
17. In the script editor, specify the name of the dataset to be used in line 4.

> load Data.mat

***Note:*** “Data.xlsx” is the name for the demonstration dataset and “Data_user.xlsx” is the name for the user-supplied dataset.

**Figure 5:**
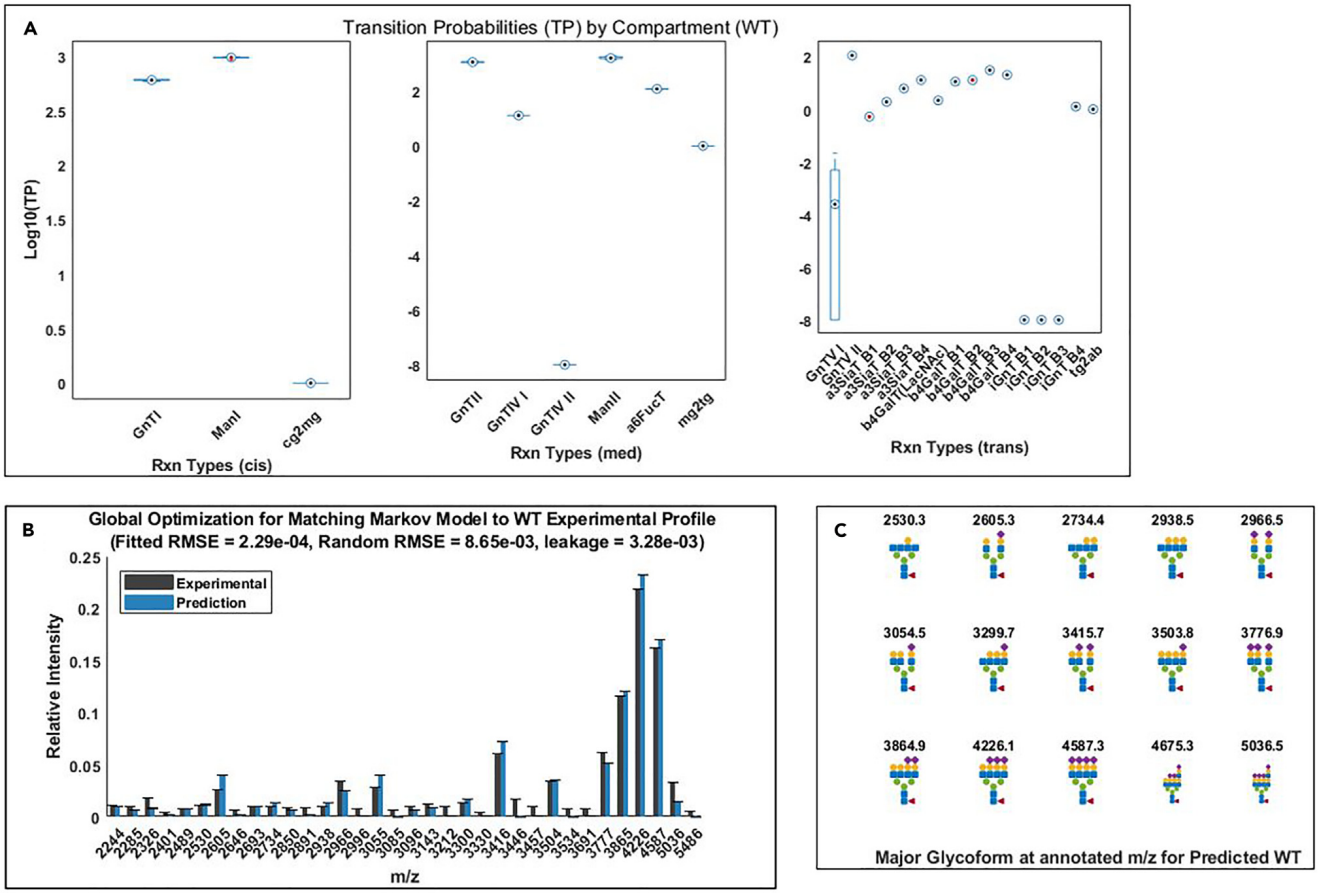
Visualization of fitted model parameters (transition probabilities) and theoretical glycoprofiles computed from the fitted Markov models for the wildtype EPO glycoprofile (A) Boxplot of the log_10_(transition probabilities) (log_10_(TP), or the model parameters) of all the fitted Markov models by reaction types. The reaction types are organized in three separate panels depending on where they primarily occur in the three different Golgi compartments represented in the models (cis, medial, and trans). The boxplot data can be inspected in the variable *Optimization.WT.xval*, where each row represents a fitted model and each column represents a reaction type in the same order of the plot’s (left to right). (B) The average of fitted glycoprofiles (Prediction) is compared with the experimental glycoprofile (Experimental). Fitted RMSE represents the root mean squared error between the fitted average and the experimentally measured signals, whereas the random RMSE represents the error between the average signals generated by multiple random models and the experimentally measured signals. Leakage represents the percentage of total signals that are not detected in the experimental measurement. Generally, well-fitted models should meet at least two criteria: Random RMSE should be at least 10 times bigger than the Fitted RMSE and the leakage should be smaller than 10%. The entirety of predicted data can be inspected in the variable *Optimization.WT.Predata_noRes*, where each column represents a m/z value (or numbering) and each row represents a fitted model. (C) The graph shows the predicted major glycoforms at the m/z of fitted signals with top relative intensities as shown in (B). These predicted glycoforms can be cross validated with existing experimental annotations if available.

**Figure 6:**
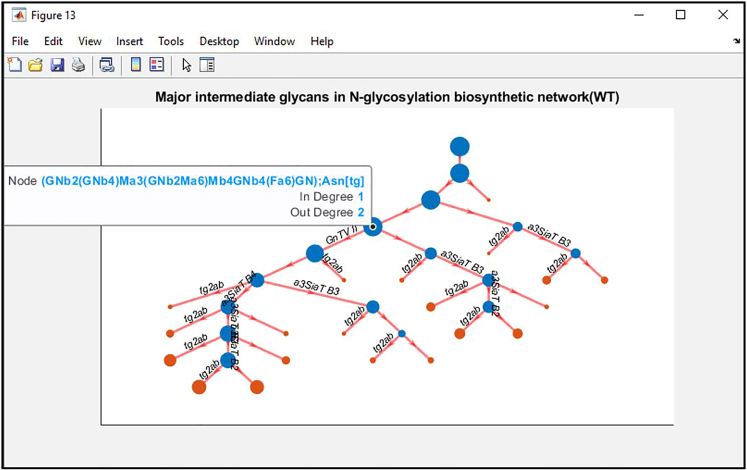
An interactive figure generated by MATLAB shows the major intermediate glycans (blue nodes) that are theoretically important to generate the experimentally observed glycans (red nodes) The size of a node is proportional to the log-scale values of all model fluxes into the node. The glycan that a node represents can be revealed by clicking the node in the MATLAB figure. Each edge represents a reaction (or reactions) required to convert one glycan to another. An edge is only labeled if there is only one reaction converting one glycan to the other.

**Figure 7:**
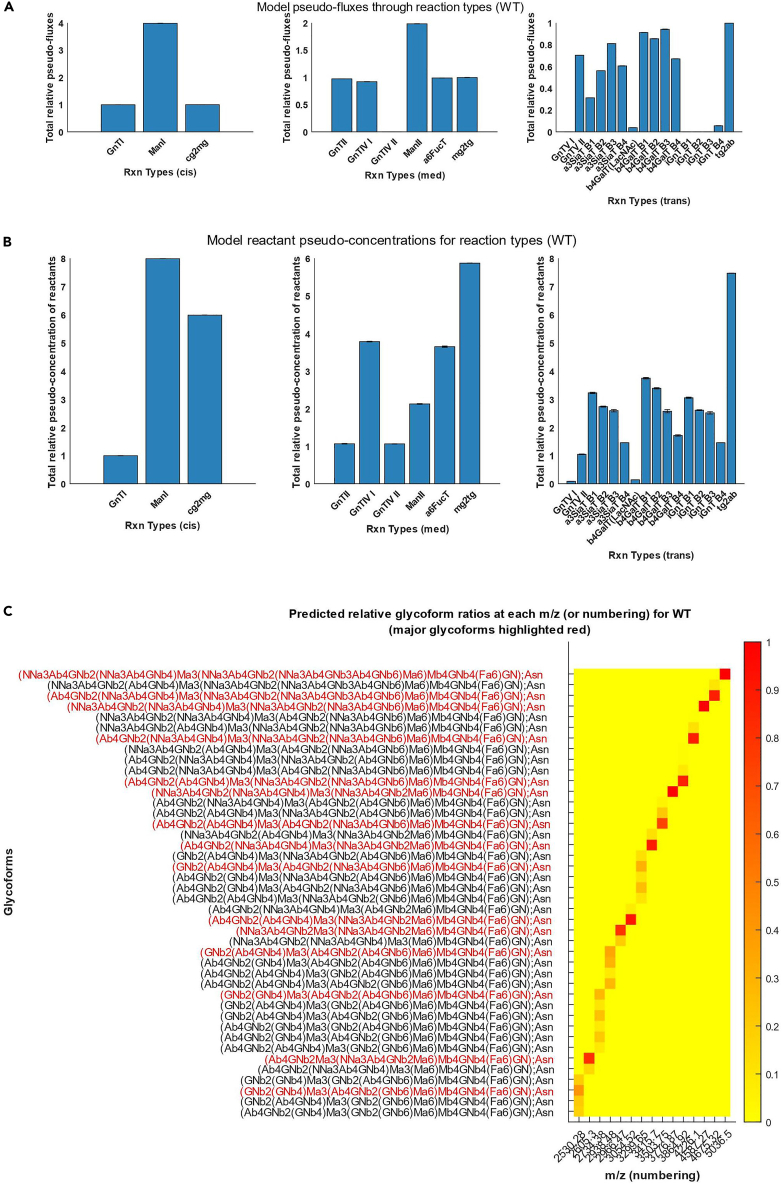
Additional network information regarding the fitted models (A) model pseudo-fluxes by reaction types and by compartments (left to right, *cis*, *medial*, *trans*-Golgi). The edges of a Markov network are associated with different reaction types, and the summations of all edge weights (models fluxes through the edges) associated with respective reaction types were defined as pseudo-fluxes. The model pseudo-fluxes can be inspected in the variable *OptimizationResults.WT.FluxesbyComp*. (B) model reactant pseudo-concentration for each reaction type and by compartments (left to right, *cis*, *medial*, *trans*-Golgi). For each reaction type, pseudo-concentration is defined as the summation of edge weights of all in-edges into the nodes that represent the glycan substrates of the reaction type. The values can be inspected by clicking the individual bars in the plot. (C) Heatmap of model predicted relative glycoform ratios at each m/z values (or numbering of glycans) based on model pseudo-fluxes. The most abundant glycoform at each m/z value is highlighted in red. Each row represents a glycoform and each column represents an m/z value. The heatmap data and the labels can be inspected in *OptimizationResults.WT.GlycoformData*.

***Note:*** Please refer to the comments in the script for additional information regarding the subfields. The new *OptimizationResults* variable is stored as *ProcessedModels.mat* in the *Data* folder.

18. Run the script and generate figures for each glycoprofile.
  **CRITICAL:** Users can assess the fitted models and glycoprofiles from their corresponding output figures. The figures generated from the fitted models of the wildtype profile (WT) are used as examples:
  a. Graphs in Figure 5 allow users to inspect the fitted model parameters (transition probabilities, or TPs) and assess the fitting quality, similarly performed in Liang et al.^1^
  b. Figure 6 allows users to inspect which glycan intermediates are theoretically important to generate the experimentally observed glycoprofiles (if the models are well-fitted).
  c. Figure 7 allows advanced users to compare additional network parameters by reaction types of the fitted Markov models.
19. Computed model features and glycoprofiles are now generated from the fitted Markov models and added as new subfields in the structure variable *OptimizationResults.*

### Predict EPO glycoprofiles following combinatorial GT knockouts from fitted EPO glycoprofiles of single GT knockouts

**Timing: 15 min**

This step predicts the EPO glycoprofiles of combinatorial GT knockouts *de novo* from the fitted EPO glycoprofiles of single GT knockouts.

20. Click on *Step5A_Conduct_Comparative_Analytics.m* in the “Current Folder” panel in MATLAB.
21. In the script editor, specify the name of the dataset to be used in line 4.

> load Data.mat

***Note:*** “Data.xlsx” is the name for the demonstration dataset and “Data_user.xlsx” is the name for the user-supplied dataset.

22. Run the script.
23. Click on *Step6A_Predict_glycoprofiles_of_combinatorial_KOs.m* in the “Current Folder” panel in MATLAB.
24. In the script editor, specify the name of the dataset to be used in line 4. “Data.xlsx” is the name for the demonstration dataset and “Data_user.xlsx” is the name for the user-supplied dataset.

> load Data.mat

***Note:*** “Data.xlsx” is the name for the demonstration dataset and “Data_user.xlsx” is the name for the user-supplied dataset.

25. Starting from Line 13, specify EPO glycoprofiles with the desired combinations of isozyme knockouts that will be predicted, along with the wildtype EPO glycoprofile.

> KnockoutSel = {{'St3gal4','St3gal6'};...

 {'Mgat2','St3gal4','St3gal6'}};

> BaseProfSel = 'WT';

***Note:*** The demonstration specifies two predictions of EPO glycoprofiles from cell lines with St3gal4/St3gal6 double knockouts and with Mgat2/St3gal4/St3gal6 triple knockouts.

26. Run the script and generate relevant figures for each predicted glycoprofile.

**Figure 8:**
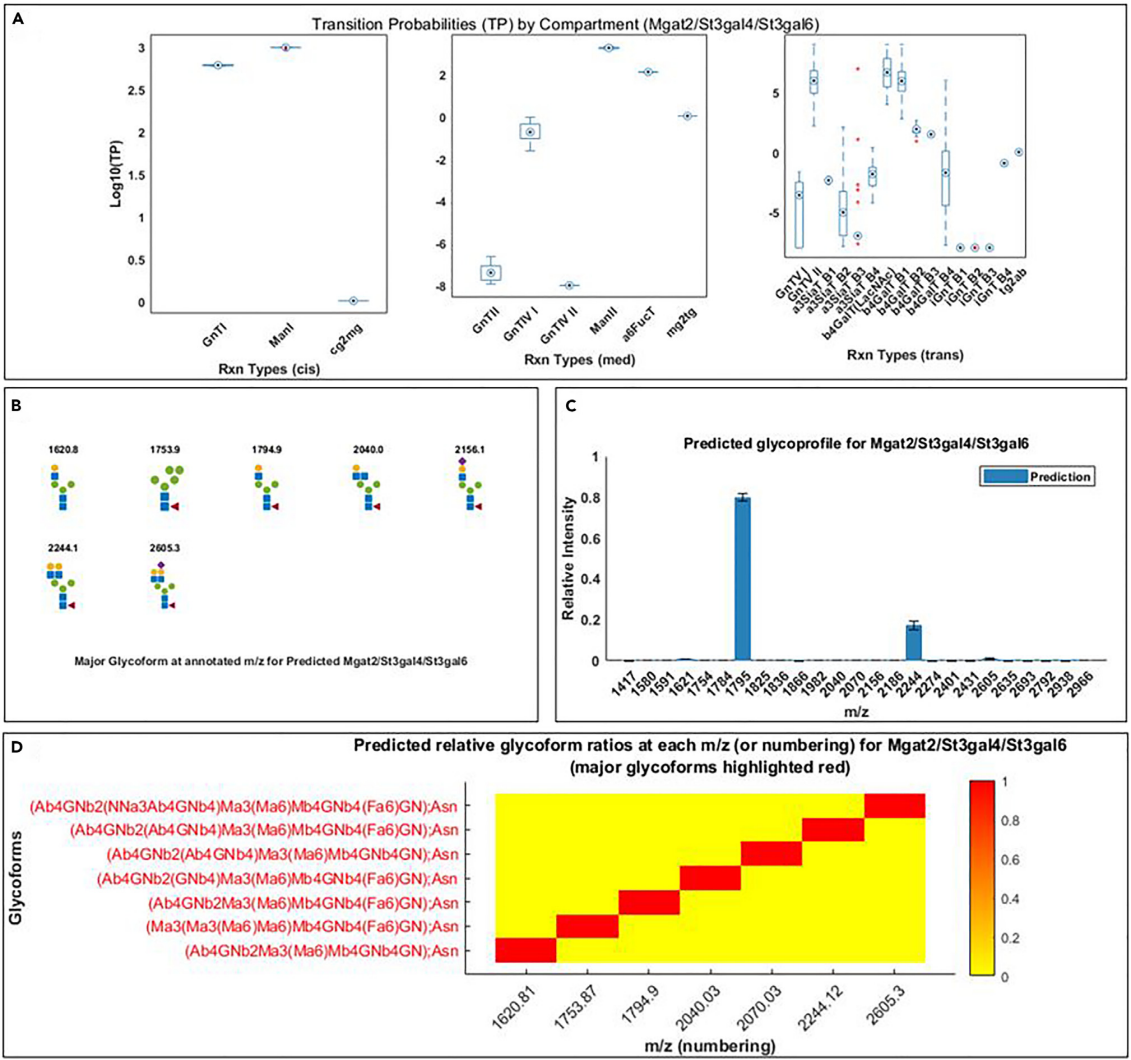
Visualization of the predicted model parameters (transition probabilities) and theoretical glycoprofiles computed from the predicted EPO glycoprofile produced by the cell line with Mgat2/St3gal4/St3gal6 triple knockouts (A) Boxplot of the log_10_(transition probabilities) (log_10_(TP), or the model parameters) of all the fitted Markov models by reaction types. The reaction types are organized in three separate panels depending on where they primarily happen in the three different Golgi compartments represented in the model (cis, medial, and trans). (B) The average of predicted glycoprofiles. (C) The graph shows the predicted major glycoforms at the m/z of predicted signals with top relative intensities shown in (B). Refer to Appendix E of Liang et al.^1^ for a complete assessment of the prediction performance of 6 different combinatorial knockouts. (D) Heatmap of model predicted relative glycoform ratios at each m/z values (or numbering of glycans) based on model pseudo-fluxes. The most abundant glycoform at each m/z value is highlighted in red. Each row represents a glycoform and each column represents an m/z value. The values of these visualized results can be found in corresponding variables detailed in the Figure 5 description.

***Note:***Figure 8 shows an example of the predicted EPO glycoprofile produced by the cell line with Mgat2/St3gal4/St3gal6 double knockouts.

### Predict Enbrel glycoprofiles from a combinatorial GT knockout, based on fitted EPO glycoprofiles of single GT knockouts and Enbrel glycoprofile produced in wildtype cells

**Timing: 12 h**

This step predicts the Enbrel glycoprofiles of combinatorial GT knockouts *de novo* from the fitted EPO glycoprofiles of single GT knockouts.

27. Following the same step for data collection (before you begin, step 10), organize the Enbrel glycoprofile produced by the wildtype cell line (as a demonstration) as the Excel file *DrugXData.xlsx.*
28. Click *Step1B_Load_Preprocessed_Data_for_WT_DrugX.m* in the “Current Folder” panel in MATLAB and run the script.
29. Click *Step2B_Create_Generic_Glycan_Network_Prediction_for_WT_DrugX.m* in the “Current Folder” panel in MATLAB and run the script.
30. Click *Step3B_Fit_Markov_Models_with_WT_Glycoprofile_for_DrugX.m* in the “Current Folder” panel in MATLAB.
31. Specify the number of models to be fitted for the wildtype glycoprofile of Enbrel in Line 17. For example:

> num = 10; % Number of models fitted for each profile

32. Specify an arbitrary number for *IDNum* in Line 31. For example:

> IDNum = 1;

> savedFileName =

 ['OptimizationResults_DrugXWT_',num2str(IDNum),'.mat'];

***Note:*** The fitted model parameters are stored in the structure variable *OptimizationResults* and as MATLAB-readable file *OptimizationResults_#.mat* in the *Data/OptimizationResults* folder. Here, number 1 is chosen and *OptimizationResults_DrugXWT_1.mat* will be the file name.

33. Run the script.

***Note:*** After step 33, users may decide whether to run multiple MATLAB sessions in parallel to reduce the time needed to obtain sufficient models for the wildtype glycoprofiles of Enbrel. The memory-intensive optimization process will need approximately 8 GB RAM per MATLAB session. The number of fitted models per hour will be approximately equal to the number of MATLAB sessions. 3 MATLAB sessions were run in parallel for this demonstration and took ∼10 h.

***Optional:*** To run a new MATLAB session in parallel, open a new MATLAB session and repeat steps 9–14. It is essential to specify a unique *IDNum* number at step 13 for each MATLAB session so that the saved files *OptimizationResults_#.mat* from the different sessions do not overwrite each other.

**Figure 9:**
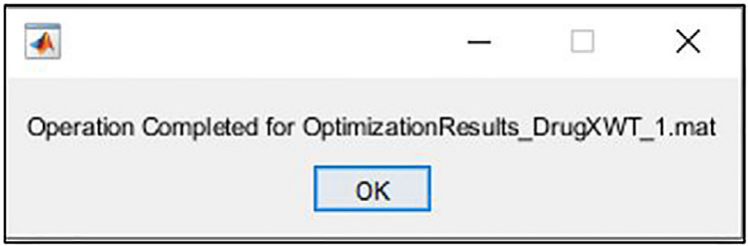
A message box will show up when fitting is completed in a ‘MATLAB session
35. Click on *Step4B_Visualize_Fitted_WT_Glycoprofiles_for_DrugX.m* in the “Current Folder” panel in MATLAB. Run the script, and figures will be generated for the fitted wildtype Enbrel glycoprofile.

***Note:*** Refer to step 18 and the descriptions of Figures 7 and 8 for the interpretation of these figures.

34. A message box (Figure 9) will appear when fitting of all the glycoprofiles in a MATLAB session is completed. If multiple MATLAB sessions are opened, close all but one MATLAB session when all runs are complete.

36. Click on and open *Step6B_Predic_glycoengineered_glycoprofiles_of_DrugX.m* in the “Current Folder” panel in MATLAB.
37. Starting from Line 13, specify EPO glycoprofiles with the desired combinations of isozyme knockouts that will be predicted, along with the name of the wildtype EPO glycoprofile.

> KnockoutSel = {{'B3gnt2'},…

 {'B3gnt2','St3gal3','St3gal4','St3gal6'}};

***Note:*** The demonstration specifies two predictions of EPO glycoprofiles from cell lines with B3gnt2 single knockout or with B3gnt2/St3gal3/St3gal4/St3gal6 quadruple knockouts.

38. Run the script, and three graphs will be generated for each predicted glycoprofile.

**Figure 10:**
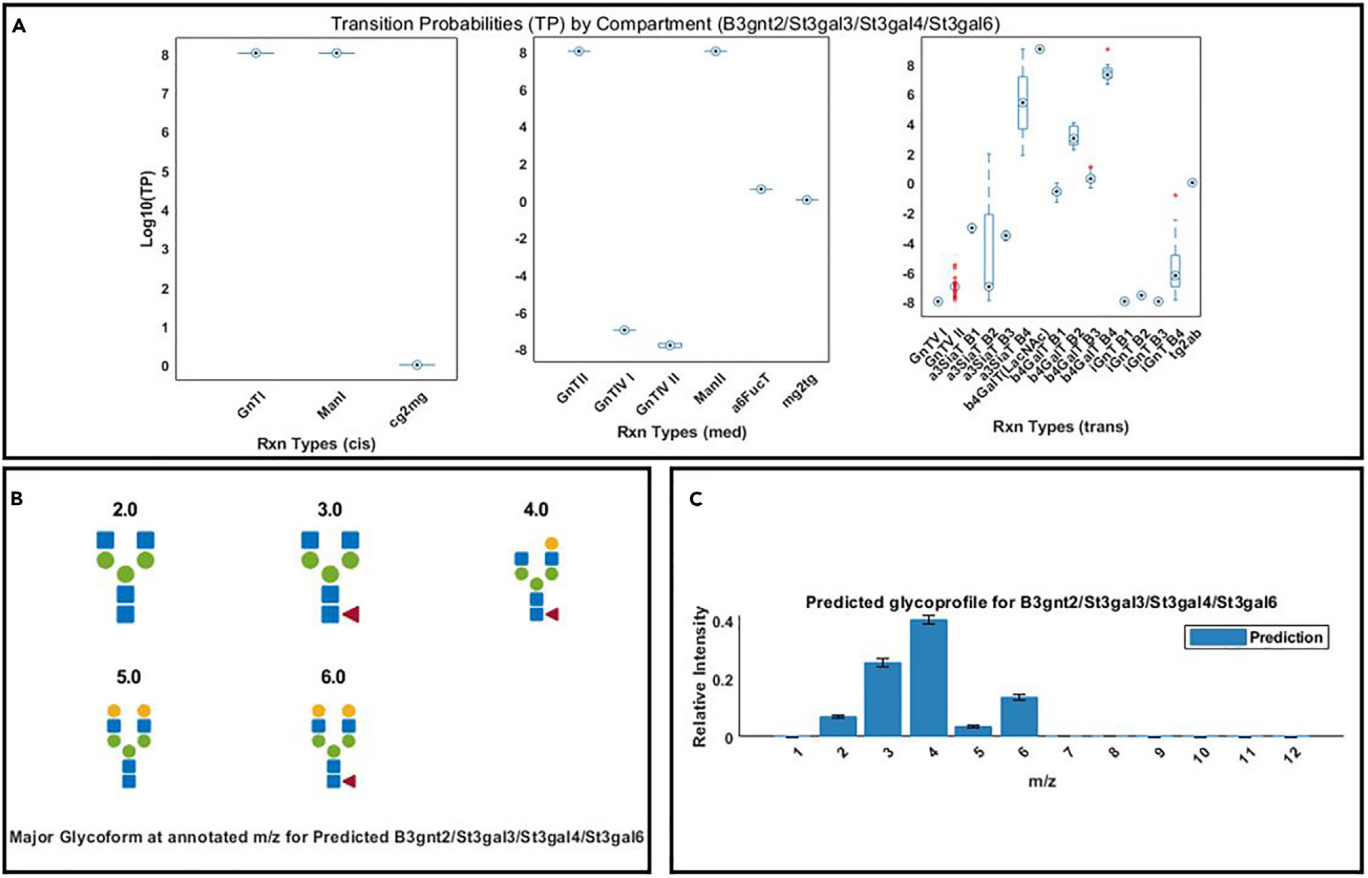
Visualization of the predicted model parameters (transition probabilities) and theoretical glycoprofiles computed from the predicted Enbrel glycoprofile produced by B3gnt2/St3gal3/St3gal4/St3gal6 quadruple knockouts (A) Boxplot of the log_10_(transition probabilities) (log_10_(TP), or the model parameters) of all the fitted Markov models by reaction types. The reaction types are organized in three separate panels depending on where they primarily occur in the three different Golgi compartment represented in the model (cis, medial, and trans). (B) The average of predicted glycoprofiles. (C) The graph shows the predicted major glycoforms at the m/z values of predicted signals with top relative intensities as shown in (B).

**CRITICAL:**Figure 10 shows an example of the predicted Enbrel glycoprofile produced by cell lines with B3gnt2/St3gal3/St3gal4/St3gal6 quadruple knockouts.

## Expected outcomes

GlycoMME is a simple-to-use yet powerful toolkit that allows users to interrogate theoretical perturbations to *N*-glycosylation biosynthesis caused by glycoengineering efforts by inferring the glycosylation processes from glycomics data. Specifically, GlycoMME is a low-parameter, biologically interpretable modeling framework that quantifies the impact of knocking out specific glycosyltransferase isozymes on the glycoprofiles of glycoproteins by modeling glycosylation as a Markov process.^1^^,^^16^^,^^20^ The framework does not rely on any manual parameter optimization or approximation of enzyme kinetics, and has demonstrated its usefulness in facilitating rational glycoengineering by predicting glycoprofiles *de novo*.^1^ Here, we demonstrated that the framework can visualize glycomic data of various resolutions (with or without glycoform annotations). The fitted results shed light on the complex interactions between glycosyltransferases and their specificities toward different *N*-glycan epitopes.^1^ GlycoMME can further use the learned impact of the GT isozymes for *de novo* prediction of glycoprofiles produced from cells with complex GT isozyme knockout genotypes, even for different recombinant glycoproteins.^1^ While shown here for the analysis of glycoengineered proteins, this framework could be used to investigate a wide range of questions, including the identification of the genetic basis of congenital disorders of glycosylation, differential regulation of glycosylation in diseases such as cancer, and a variety of other biological questions involving changes in glycan structures.^23^^,^^24^^,^^25^^,^^26^

Given that the model parameters are associated with specific glycosyltransferase reactions, GlycoMME has the potential to generate hypotheses directly testable by other omics data, such as proteomics and transcriptomics. The integration of glycomics with other omics data in the framework of GlycoMME will help us decipher the complex regulatory machinery of *N*-glycosylation and make rational glycoengineering a near possibility.

## Limitations

While GlycoMME can accurately reproduce a variety of glycoengineered glycoprofiles, the real biological system of *N*-glycosylation is more complicated than the Markov model in its current form. Evidently, fitted models are in general more accurate than predicted models, and some fitted glycoprofiles are more accurate than others. Therefore, errors may be expected when predicted glycoprofiles are generated under different conditions from those of the fitted glycoprofiles, such as variations in cell types, media/supplement, and other genetic manipulations that may indirectly influence GT activities in *N-*glycosylation. More research is needed to improve model parametrization and make GlycoMME more versatile in considering glycoengineering impact beyond GT knockouts.

## Troubleshooting

### Problem 1

At step 3, MATLAB returns an error message and Figure 3B cannot be generated even though there are experimental glycan annotations entered in the *Data.xlsx* or *Data_user.xlsx* file.

### Potential solution

There may be an error in the glycan annotations entered in the *Data.xlsx* or *Data_user.xlsx* file. Please double check glycan annotations entered in the second column in the sheet “Annotation”. Also check if there are any identical annotation strings in the column.

### Problem 2

While running the script *Step4A_Visualize_Fitted_Glycoprofiles.m* or *Step4B_Visualize_Fitted_WT_Glycoprofiles_for_DrugX.m* (steps 18 or 35), MATLAB returns an error message indicating “Error in PlotTPbyComp (line 54)”

### Potential solution

The error is caused by inability to reconstruct the models from the provided model parameters. First, the user should ensure that the variable *OptimizationResults.Name.xval* is not empty. Users may check directly by typing “isempty(OptimizationResults.(ProfSel{a}).xval)” in the command window. If the variable is indeed empty, change the value of Method (line 48) from “'KernalDensity” to “Outlier” for the function *FilterOptimizationResults* and rerun the entire script. If the issue is still not resolved, obtain additional models by rerunning the fitting steps for the specific profile from steps 9–19.

### Problem 3

At steps 18 or 35, the fitting quality is not satisfactory, such that the leakage is greater than 0.15 and/or the ratio of random RMSE/fitted RMSE is less than 5 (Figure 5B).

### Potential solution

Double check the grammar of the Linear Code and the composition strings for this profile in the “Annotation” or “MS Raw” sheets of the source data file (e.g., *Data_user.xlsx*), especially for signals with significant errors between the experimental and the fitted profiles. Upon confirmation, obtain additional models (>6 models) by rerunning the fitting steps for the specific profile from steps 9–19.

### Problem 4

At steps 18 or 35 (Figure 5B), the uncertainties of the top signals (>5%) are very large (signals >0.05, and error bars >30% of the signals).

### Potential solution

If annotation exists for these signals, recheck the corresponding glycoforms of their annotations (Figure 1B). Upon confirmation, change their values from 1 to 0 in the “Annotation” sheet of the source data file (e.g., *Data_user.xlsx*). Rerun steps 1–8. Next, type “open SetUpFittingProblem” in the command window and change the value for “MaxTime” from 3600 to 7200 at line 70 of the opened script. Save the modified script. Finally, obtain additional models (>6 models) by rerunning the fitting steps for the specific profile from steps 9–19.

### Problem 5

MATLAB returns an error message “Unrecognized field name “XX".” at step 26, where “XX” is an entered profile name.

### Potential solution

This error occurs when the enzyme names used for knockout annotations are inconsistent between the single-knockout models and the predicted multiple-knockout models. Ensure to only use alphanumerical characters for knockout profile names (e.g., the knocked-out enzymes’ names) without space. Upon confirmation, ensure the variables *KnockoutSel* and *BaseProfSel* modified at step 25 are consistent with the names of the fitted profiles.

## Resource availability

### Lead contact

Further information and requests for resources and reagents should be directed to and will be fulfilled by the lead contact, Nathan E. Lewis, (nlewisres@ucsd.edu).

### Materials availability

This study did not generate new unique reagents.

### Data and code availability

The EPO datasets were obtained from a previous study^22^ and processed to be used as a part of the example dataset. The processed example datasets and the pipeline codes are available at https://github.com/LewisLabUCSD/N-Glycosylation-Markov-Models/tree/main (https://doi.org/10.5281/zenodo.7742912). The complete Enbrel dataset supporting the current study has not been deposited in a public repository because of ongoing investigation of the dataset but is available from the corresponding author on request.
